# Addressing child protection issues in Bangladesh's Rohingya and host community to improve children's health

**DOI:** 10.1016/j.lansea.2022.100070

**Published:** 2022-09-06

**Authors:** Zeeba Zahra Sultana, Adittya Arefin, Ahmed Hossain

**Affiliations:** a Global Health Institute, North South University, Dhaka 1229, Bangladesh; b Department of Health Services Administration, University of Sharjah, United Arab Emirates

The Rohingya refugee crisis has changed Cox's Bazar-Teknaf peninsula in immense ways over the last five years in Bangladesh. Currently, it is hosting approximately 0.3 million children aged 5–17 years, which is 68.5% of the Rohingya children (0–17 years).^1^ Violence in Myanmar disrupted the identities of the Rohingya people, including their education, livelihoods, and family lives, and rebuilding these has been challenging, particularly for children, as they face new challenges in their surroundings. According to UNICEF, the key monitoring indicators of child protection issues are birth registration, child labour, child marriage, female genital mutilation, and violence against children.^2^ We aim to understand birth registration, child labour, child marriage, and educational achievement in the Rohingya and host communities while relating these child protection issues to the children's health.

Because of the influx in 2017 of Rohingya migrants, the regular child registration process in Cox's Bazar was halted until September 2020.^3^ The aim was to prevent Bangladeshi birth certificates from being issued to Rohingya through fraudulent routes. Moreover, it is a common practice for host families to register births right before the child begins school. The children that currently need schooling are typically affected by this temporary suspension and late registration, which increases their vulnerability to sending the boy child to paid work and marrying off the girl child. According to a recent study, child labour increased by 49% of host households and 16% of Rohingya households during the COVID-19 pandemic, while underage female marriage increased by 20% and 9% in Rohingya and host households, respectively.^4^ Additionally, it was found that 16% of host families and 2% of Rohingya families reported missing children, many of whom were engaged in paid work away from home.^4^

Myanmar's government prohibited underage marriage; even legal marriages between Rohingya men and women required permission. Following their relocation to Bangladesh, they were no longer subject to such restrictions. According to a recent survey, 9.4–14.3 percent of Rohingya girls marry before they reach the age of 18.^5^ Notably, in the refugee population, 31.5% of children are under the age of five, implying that many children were born after the forced exodus in 2017.^1^ Girls in refugee camps are at risk of marriage as soon as they reach puberty if no special effort is made to protect them. As a result of financial pressures on families or the widespread assumption that a girl must get married to be safe, this is becoming increasingly common in communities.

Table 1 provides a summary of three child protection issues. There is a data gap concerning the employment of refugee children, as seen in the table. Moreover, there is a lack of access to education in the Rohingya language, including skill-building initiatives, making them vulnerable to child labour or marriage. Before the COVID-19 outbreak, over 78% and 14.8% of children between the ages of 6–14 years and 15–18 years reported going to a temporary learning facility, respectively.^4^ The startling fact is that only 3% of adolescent Rohingya girls participated in academic programs.^4^ It can partly be explained by the fact that when Rohingya girls reach adolescence, parents typically drop them out of school to avoid interacting with men to ensure security, protect traditional norms, and help the family at home. On the other hand, the percentage of children (5–17 years) going to school ranges from 73% to 82% in the host community.^4^

**Table 1:** Descriptive statistics of the child protection issues.

Traits	Rohingya	Host community (People live next to Rohingya camps)	Bangladesh
Working children	–	3% (Working children)^4^	4.41% (Working children) 4.28% (child labour)^7^
Women married before age 18/Currently married	9.4% (15–17 years) 13.5% (18–24 years)^5^	–	31.9% (15–19 years) 51.4% (20–24 years)^6^
Children attending school	61.64%^a^ (6–17 years)^4^	73–78% in male 75–82% in female (5–17 years)^4^	88.40% (5–14 years)^6^

a Percentage of children attending a temporary learning centre.

In studies examining the impacts on mental health in a refugee context, it was found that adolescents who engaged in paid work or got married had high psychological symptoms.^8^^,^^9^ In addition to violating human rights, child marriage raises the risk of depression, STDs, cervical cancer, obstetric fistulas, and maternal death. Moreover, high school dropouts may increase criminal activity, drug use, long-term health problems, and mental illness. A survey shows over half of Rohingya children suffer from emotional disorders.^10^ Children from the Rohingya community are the most affected by problems with poor child protection, and more must be done to enhance their health before the effects of their plight as refugees worsen.

To effectively improve the health of children, a robust situation analysis of the protection issues and their causative factors is urgently required. Resilient public-private partnerships are needed to completely prohibit child marriage and labour, ensure education till eighth grade (a law in Bangladesh), engage communities for unreported birth registration, raise awareness and educate parents regarding the stages of cognitive development of a child and the effect, and consequence of any disruption.

## Contributors

ZZS, AA and AH contributed equally to literature search, writing, editing and approved the final manuscript.

## Declaration of interests

The authors declare no known competing financial interests or personal relationships that could have appeared to influence the work reported in this paper.

## References

[1] Government of Bangladesh/UN High Commissioner for Refugees, Joint Government of Bangladesh - UNHCR Population factsheet - block level as of 31 March 2022. Available:https://reliefweb.int/report/bangladesh/joint-government-bangladesh-unhcr-population-factsheet-block-level-31-march-2022. Accessed 24 June 2022.

[2] UNICEF 2015, Child protection overview. Available: https://data.unicef.org/topic/child-protection/overview/. Accessed 24 June 2022.

[3] Assessment Capacities Project 2021, Bangladesh: Impact of the suspension of birth registration on the host community in Cox's Bazar, Thematic Report, 3 August 2021. Available:https://reliefweb.int/report/bangladesh/bangladesh-impact-suspension-birth-registration-host-community-cox-s-bazar. Accessed 24 June 2022.

[4] Inter Sector Coordination Group, Joint Multi-Sector Needs Assessment - Rohingya Refugee, May, 2021. Available:https://reliefweb.int/report/bangladesh/joint-multi-sector-needs-assessment-j-msna-bangladesh-rohingya-refugees-may-2021. Accessed 24 June 2022

[5] PB Jennifer Leigh Alexa Edmier, Janna Metzler, Courtland Robinson, Thakshayeni Skanthakumar, “Child Marriage in Humanitarian Settings in South Asia: Study Results from Bangladesh and Nepal. UNFPA APRO and UNICEF ROSA.,” 2020. Available:https://www.unicef.org/rosa/reports/child-marriage-humanitarian-settings-south-asia. Accessed 24 June 2022

[6] Bangladesh Bureau of Statistics (BBS) and UNICEF Bangladesh. 2019. Progotir Pathey, Bangladesh Multiple Indicator Cluster Survey 2019, Survey Findings Report. Dhaka, Bangladesh: Bangladesh Bureau of Statistics (BBS). Available:https://www.unicef.org/bangladesh/media/3281/file/Bangladesh%202019%20MICS%20%20Report_English.pdf. Accessed 26 June 2022

[7] ILO report 2015, Bangladesh National Child Labor Survey 2013.Available: https://www.ilo.org/ipec/Informationresources/WCMS_IPEC_PUB_28175/lang–en/index.htm. Accessed 26 June 2022

[8] Meyer SR, Yu G, Rieders E, Stark L. (2020). Child labor, sex and mentalhealth outcomes amongst adolescent refugees. J Adolesc.

[9] Hossain A, Ahmed S, Shahjalal M, Ahsan GU. (2019). Health risks of Rohingya children in Bangladesh: 2 years on. Lancet.

[10] Khan NZ, Shilpi AB, Sultana R (2019). Displaced Rohingya children at high risk for mental health problems: findings from refugee camps within Bangladesh. Child Care Health Dev.

